# Demonstration of an integrated nanophotonic chip-scale alkali vapor magnetometer using inverse design

**DOI:** 10.1038/s41377-021-00499-5

**Published:** 2021-03-11

**Authors:** Yoel Sebbag, Eliran Talker, Alex Naiman, Yefim Barash, Uriel Levy

**Affiliations:** Department of Applied Physics, The Center for Nanoscience and Nanotechnology, The Hebrew University of Jerusalem, Jerusalem, 91904 Israel

**Keywords:** Optical metrology, Integrated optics, Atom optics

## Abstract

Recently, there has been growing interest in the miniaturization and integration of atomic-based quantum technologies. In addition to the obvious advantages brought by such integration in facilitating mass production, reducing the footprint, and reducing the cost, the flexibility offered by on-chip integration enables the development of new concepts and capabilities. In particular, recent advanced techniques based on computer-assisted optimization algorithms enable the development of newly engineered photonic structures with unconventional functionalities. Taking this concept further, we hereby demonstrate the design, fabrication, and experimental characterization of an integrated nanophotonic-atomic chip magnetometer based on alkali vapor with a micrometer-scale spatial resolution and a magnetic sensitivity of 700 pT/√Hz. The presented platform paves the way for future applications using integrated photonic–atomic chips, including high-spatial-resolution magnetometry, near-field vectorial imaging, magnetically induced switching, and optical isolation.

## Introduction

Over the last few years, there has been growing interest in developing quantum integrated systems and miniaturizing rubidium cells ranging from the centimeter scale to the micro- and nanoscale. A significant step forward is achieved by combining alkali vapor with guided wave configurations like photonic crystal fibers^1,2^, antiresonant waveguides^3^, and tapered fibers^4,5^, and with nanoscale photonic structures, including nanowaveguides^6–8^, resonators^9,10^, and nanoantennas^11^. In addition to the obvious advantages of such integration, other great qualities result from this approach. For example, tight confinement of light in a nanoscale waveguide leads to strong atom-light interactions, and enhanced nonlinear processes. This extreme confinement allows observation of significant nonlinear effects at very low optical powers (nanowatts), paving the way for applications like few-photon communication systems based on all-optical switching^8^. Another example showing the advantages provided by integrated systems is the recent demonstration of an efficient optical isolator based on transverse photon spin and linear momentum locking of the evanescent field interacting with rubidium (Rb) vapor^12^.

The capabilities of chip-scale nanophotonic components can be fully exploited by advanced techniques such as computer-assisted algorithms that enable optimization of the design for a given performance envelope. For example, in inverse design^13^, the desired specifications of the device are defined as input, and an optimization algorithm searches for the best topological structure that meets the requirements. The design flexibility offered by such techniques can benefit the development of miniaturized alkali-based quantum sensors.

One of the major applications of Rb vapor is magnetometry. Optical magnetometers based on alkali vapor are among the most sensitive devices for measuring magnetic fields. Such devices generally consist of a glass cell containing a vapor of alkali atoms, such as rubidium or cesium, interacting with resonant light probing the coherent precession of polarized atomic spins, which is directly proportional to the surrounding magnetic field to be measured. Recent developments in this field have led to significant improvements, reaching sensitivities in the sub-fT Hz^−1/2^ range^14,15^. The interest in highly sensitive optical magnetometers is driven by numerous and diverse applications, including testing of the fundamental symmetries of nature, dynamic measurement of biomagnetic fields, sensing of magnetic micro-particles at ultra-low concentrations, signal detection in NMR and MRI, direct detection of magnetic fields from the heart and the brain, or magnetic microscopy^15,16^.

The fundamental quantum-mechanical uncertainty in the measurement of the atomic spin projection is given by the atomic shot-noise-limited magnetic sensitivity δ*B*_ASN_ of a polarized atomic sample. It is determined by the total number of atoms *N* and the spin-relaxation rate Γ, and for the case of measurement times $\tau \gg {{\Gamma }}^{ - 1}$^16,17^:

1 $$\delta B_{\mathrm{ASN}} \approx \frac{1}{\gamma }\sqrt {\frac{{\Gamma }}{{N\tau }}}$$

where *γ* is the gyromagnetic ratio of the atomic species.

In miniaturized vapor cells, the magnetic sensitivity rapidly decreases with the volume of the atomic vapor cell since wall collisions of the moving atoms significantly increase the spin-relaxation rate. Therefore, in practice, the tradeoff between sensitivity and resolution limits the spatial resolution of these highly sensitive magnetometers, and the typical interaction volume ranges from 1 cm^3^ to a few mm^3^ ^16,18^.

Further miniaturization and integration of alkali-based magnetic sensors on-chip using conventional integrated photonic structures is challenging. Many highly sensitive magnetometers depend on the ability to precisely monitor and control the state of polarization of the light interacting with the atoms. In integrated waveguides, magneto-optic rotation requires phase matching between the fundamental transverse electric (TE) and transverse magnetic (TM) modes^19^, which is difficult to obtain in highly confined asymmetric waveguides^20^.

**Fig. 1:**
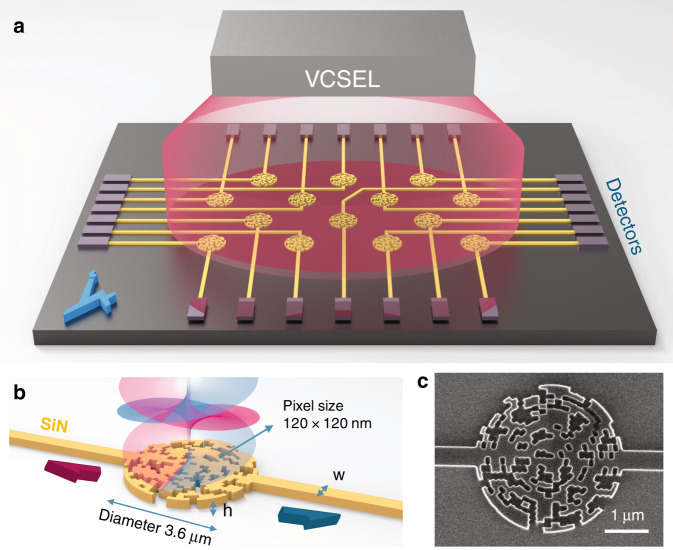
PSS concept and design. **a** Conceptual sketch of an integrated magnetic sensor array. **b** Schematic illustration showing the operation of the photonic spin selector, presenting the details of the subwavelength structure. The width, *w*, is 450 nm, and the height, *h*, is 250 nm. **c** Scanning electron microscope (SEM) image of the fabricated device with a scale bar of 1 μm.

In this work, we propose and experimentally demonstrate a nanophotonic Rb-based magnetic sensor with high-spatial resolution. This is achieved by integrating a 30 μm microfabricated Rb cell into an integrated photonic spin selector (PSS) that is implemented using an inverse design method. The PSS consists of a circular-shaped structure with subwavelength features, with two single-mode waveguide outputs. It is designed to spatially resolve the handedness of incident photons such that the information on the atomic spin precession can be easily retrieved and analyzed. The development of chip-scale integrated magnetic sensors based on alkali vapor opens the path towards implementing 2D magnetic sensors presenting both high magnetic sensitivity and high-spatial resolution. The conceptual vision of 2D mapping of the magnetic field using the presented integrated sensor array is depicted in Fig. 1a. As presented in this sketch, each PSS can be viewed as a single pixel measuring the magnetic field. The spatial resolution is defined by the geometry of the PSS and the volume of the integrated Rb vapor cell above it.

## Results

### Approach to design an integrated quantum magnetic sensor

There are many configurations for optical magnetometers. The two most common detection schemes are based on monitoring either the polarization of transmitted light or its intensity. The simplest scheme to implement in miniaturized optical magnetometers is based on absorption measurements, such as in Mz^21^, Mx^22,23^, or frequency modulated Bell–Bloom schemes^24^. In these schemes, pumping and probing are performed with the same beam, and the magnetic field is detected as a change in the intensity of the probe light. A sensitivity as low as 5 pT/√Hz^22^ was experimentally demonstrated in microfabricated Rb cells using this technique.

The second scheme, based on nonlinear magneto-optical rotation (NMOR), allows the development of highly sensitive magnetometers with sensitivities as low as a few femtoteslas^25^, even for millimeter-long Rb vapor cells. Magnetometers based on NMOR rely on the precise measurement of the state of polarization of the light interacting with the atoms as a function of the magnetic field strength. Measurement of polarization rotation, i.e., the magnetically induced circular birefringence, is straightforward in free-space configuration, using a half-wave plate prior to a polarization beam splitter. An equivalent way of tracking the change of polarization is to measure the modification of ellipticity induced by magnetically dependent circular dichroism, by differentiating the relative quantities of right and left circularly polarized photons^26^. This can be done by designing a photonic device acting as a photon spin selector, spatially resolving the handedness of the incident photons.

Hereby, such a device is implemented using the inverse design method in which a photonic structure is generated by an algorithm optimizing a user-defined cost function. To generate an efficient PSS, a homemade algorithm based on the direct-binary-search (BDS) algorithm^27^ was implemented in MATLAB, combined with three-dimensional finite-difference time-domain (FDTD) simulation (Lumerical). The device was fabricated using a standard silicon wafer with a 250-nm-thick layer of silicon nitride (SIN) grown on top of a 2 μm layer of thermal oxide. The device area consisted of a miniaturized circular-shaped structure with a radius of 1.8 μm, and the whole area was divided into 450 subwavelength pixels. The interval in the radial direction was ~120 nm, while the azimuthal interval varied from 120 to 190 nm to keep the number of pixels reasonable. For each pixel, the material could be either silicon nitride or air, as determined by the optimization algorithm. The figure of merit (FOM) for the optimization was defined to maximize the coupling efficiency of right circularly polarized light to the fundamental TE mode of a waveguide and left circularly polarized light to the fundamental TE mode of another waveguide (Supplementary Information 1). The dimensions of the optimized PSS and a scanning electron microscope image of the fabricated device are presented in Fig. 1b, c, respectively.

### Integrated photonic spin selector

**Fig. 2:**
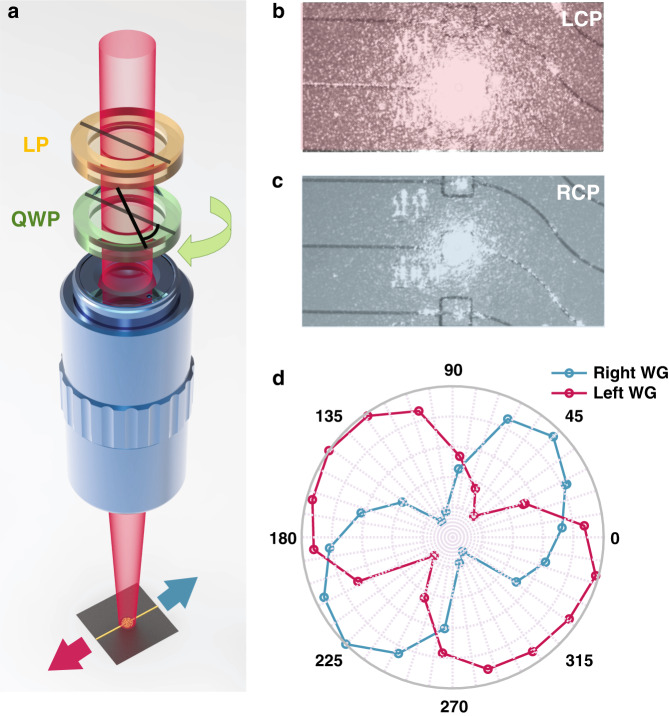
PSS characterization. **a** Sketch of the characterization setup. A laser beam is focused on the fabricated device, while the incident polarization is scanned using a rotating QWP. The intensity outcoupled from the different waveguides is monitored with respect to the QWP angle. LP linear polarizer. QWP quarter-waveplate. **b** Microscope image of the scattered light coupled to a waveguide when the incident laser beam is left circularly polarized (LCP) and **c** right circularly polarized (RCP). **d** Normalized light intensity collected from each waveguide as a function of the relative angle between the fast axis of the QWP and the LP.

The sorting performance of the device was characterized by performing two types of measurements. First, a laser beam at a wavelength of 780 nm was focused on the PSS. The incident light polarization was defined by a linear polarizer (LP) and a quarter-waveplate (QWP). By rotating the angle of the QWP with respect to the LP, the incident polarization was tuned from circular to linear, passing through elliptical. The light was then coupled to the output waveguides, as shown in Fig. 2a. When the QWP is at 45° with respect to the LP, the incident light is right circularly polarized, and the light is more efficiently coupled to one waveguide, while at an angle of 135°, the incoming light is left circularly polarized and couples to the other waveguide. Microscope images presenting the collected scattered light are presented in Fig. 2b, c. The output intensities collected from both waveguides are presented in Fig. 2d, as a function of the angle defined by the axes of the QWP and the LP. The outputs show a typical sin^2^(θ) dependency, as expected from the Jones calculus. For circularly polarized incident light, the contrast, defined as the relative difference between the outputs of the two waveguides, was measured to be ~88 ± 2%, and the coupling efficiency was measured to be ~1%. These numbers were found to be in good agreement with the simulation, presenting a contrast of 95% and a coupling efficiency of 1.5% (Supplementary Information 1).

**Fig. 3:**
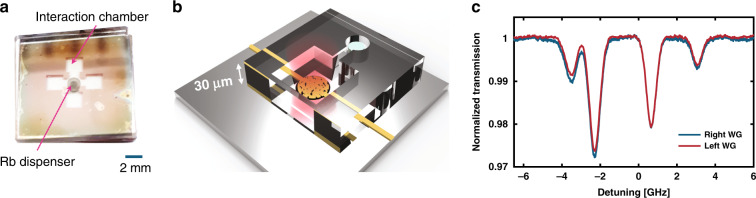
Micrometer Rb cell. **a** Microscope image of the micrometer Rb cell, with a scale bar of 2 mm. The cell consists of a central chamber containing a Rb dispenser pill and four clear interaction chambers. Each chamber is 2 mm × 2 mm wide and 30 µm deep. **b** Sketch of the device presenting the micrometer Rb cell implemented on top of the photonic chip. The PSS is depicted in yellow, to which an incident laser beam is coupled. **c** Spectra of the miniaturized Rb cell, as collected from the waveguides, without any applied magnetic field, showing degenerate spectra at the outputs of the right and left waveguides.

In the second experiment, we demonstrated how the PSS can be used to spatially resolve the information contained in different handedness chiral light-matter interactions due to the magneto-optic effect in a microfabricated Rb vapor cell. The cell consists of two patterned borosilicate glass wafers bonded by anodic bonding technique^28^ in a custom-made vacuum chamber. The cell contains a hole for an Rb dispenser pill and 30 μm deep Rb interaction chambers, which are directly etched into the glass (see “Materials and methods” section for more information about the fabrication procedure). The dimensions of the device are presented in Fig. 3a. The microfabricated cell was then deposited on top of the PSS, and linearly polarized light was focused on the PSS. In the future, the cell can be directly bonded to the photonic chip by silicon-to-glass anodic bonding^29^. Before characterizing the chiral discrimination properties of the PSS, we first characterized the microfabricated Rb cell implemented on the PSS. In the experimental setup depicted in Fig. 3b, a linearly polarized scanning laser beam resonant with the D2 line of Rb was focused on the PSS after passing through the vapor cell, which was heated to ~70 °C. In the spectroscopy measurement presented in Fig. 3c, the outputs of both waveguides present the same response.

**Fig. 4:**
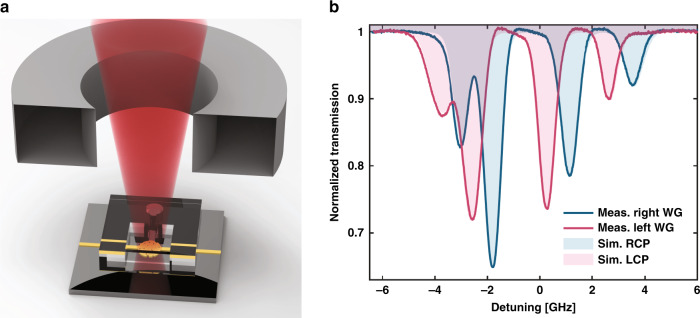
Circular dichroism in the micrometer atomic chip. **a** Sketch of the experimental setup presenting the neodymium magnet on top of the device and linearly polarized laser beam focused on the PSS. **b** Normalized intensity collected from each waveguide, presenting different spectra for LCP and RCP light spatially resolved by the PSS. The fitted simulated spectra for interaction with a 30-µm-long cell under a uniform magnetic field of 250 G are also presented as the shaded region, showing an excellent fit to the experimental data.

Following this experiment, a neodymium permanent magnet was bought close to the cell, as depicted in Fig. 4a, applying a nearly uniform magnetic field of ~250 G on the Rb vapor, which was heated to ~120 °C. This magnetic field induces Zeeman splitting of the atomic transitions such that a different absorption spectrum is experienced by the interacting photons depending on their spin. Then, the PSS spatially resolves the incident photons depending on their spin and couples them to their corresponding waveguide. The spectra measured at the outputs of both waveguides are presented in Fig. 4b, showing that the information imprinted on each photonic spin is efficiently discriminated. The graph also presents the theoretical curves obtained by solving the optical Bloch equations. The difference in the observed spectrum for each circular polarization is expected due to the different coefficient strengths for each transition^12,30^.

### Magnetic-field measurements

**Fig. 5:**
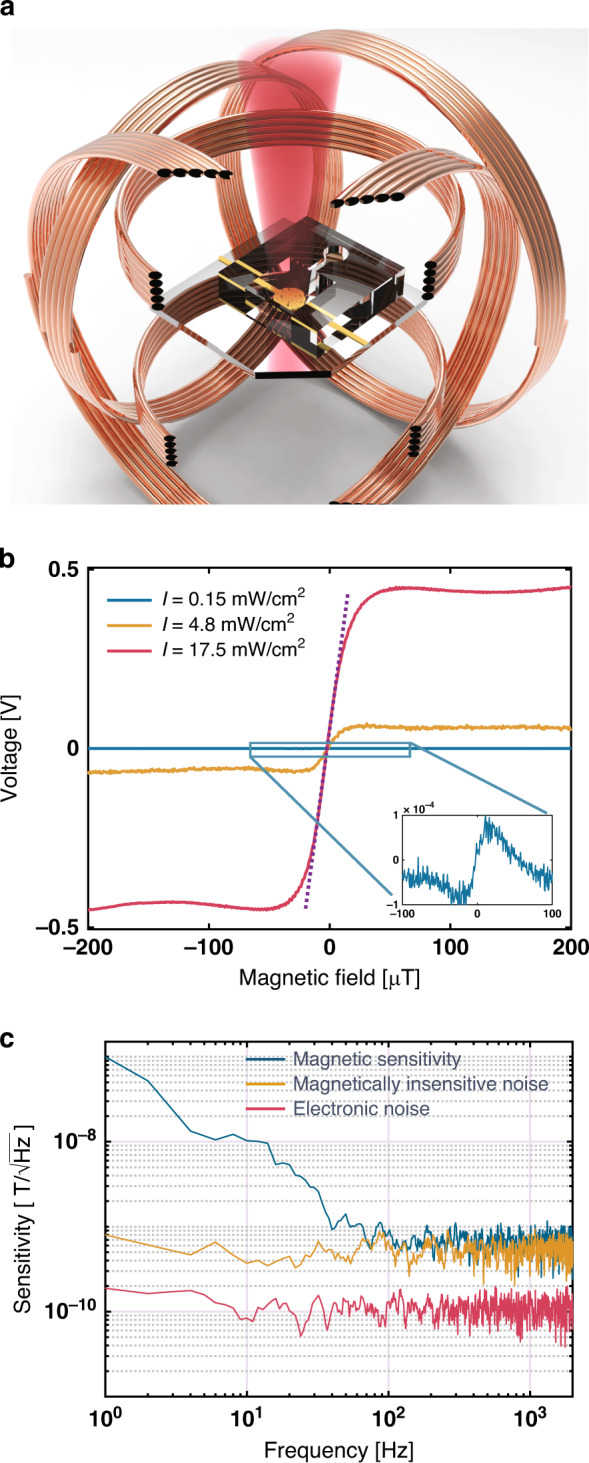
Magnetic-field measurements. **a** Sketch of the experimental setup presenting the device inside a set of Helmholtz coils used for the magnetic measurements. **b** NMOR signal measured for different light intensities. **c** Magnetic sensitivity measurement showing the measured magnetic sensitivity (blue) for the case of light intensity of 17.5 mW/cm^2^, magnetically insensitive noise (orange) measured by far detuning the laser frequency from the Rb transition, and electronic noise (red).

Next, we turn to magnetic-field measurements. A schematic sketch of the experimental apparatus is shown in Fig. 5a. The atomic chip was placed in the center of a set of custom-made Helmholtz coils to null the surrounding magnetic field and heated to ~120 °C. This temperature was chosen considering the tradeoff between increasing the density of the Rb atoms and minimizing the spin destruction induced by Rb collisions (Supplementary Information 2 and 3). An additional pair of coils were added to scan the magnetic field along the propagation direction of the light. A nearly resonant linearly polarized laser beam was focused, though the cell, on the PSS. The optical signals at the outputs of both waveguides were collected by two lensed fibers and detected by a balanced photodetector. To compensate for the different coupling efficiencies from each waveguide to the fibers, the relative intensity in each channel was adjusted with a variable attenuator fiber prior to the detector such that without the application of a magnetic field, the voltage on the balanced photodetector was zero. Figure 5b shows the typical dispersive shape of the NMOR response for different laser intensities as a function of the magnetic-field component parallel to the light propagation direction, *B*_z_. The laser frequency was detuned by 200 MHz from the 5^2^S_1/2_(*F* = 3) → 5^2^P_3/2_(*F* = 4) hyperfine transition of ^85^Rb. The linear slope of the dispersive lineshape increases with the laser intensity, as expected from NMOR^31–33^. At an intensity of ~17.5 mW/cm^2^, the detected signal depends linearly on the field in the range of approximately ±10 μT, which practically determines the dynamic range of the magnetometer.

We evaluated the performance of the magnetometer at a laser intensity of 17.5 mW/cm^2^ by monitoring the noise level at the output of the balanced photodetector while all three components of the background magnetic field were zeroed. The resulting spectra are shown as solid lines in Fig. 5c. For noise frequencies between dc and ~40 Hz, we observe a typical 1/*f* behavior of the magnetic noise that is attributed to fluctuations in the ambient magnetic field. It is due to either imperfect magnetic nulling or fluctuations in the compensation coil current sources. At higher frequencies, the noise floor, determining the sensitivity of the magnetometer, was measured to be ~700 pT/√Hz. In this experiment, the photon shot-noise-limited sensitivity was estimated to be ~80 pT/√Hz (using an intensity value of 17.5 mW/cm^2^), and the higher value of the measured noise floor can be attributed to issues such as laser intensity noise, fluctuations in the coupling efficiency between the photonic chip and the lensed fibers due to mechanical vibrations and electronic noise. This system noise is presented as the magnetically insensitive noise spectrum. It was obtained by far detuning the laser frequency by a few tens of GHz from the Rb resonance, where the magneto-optic effect in Rb is negligible, while all parameters were kept unchanged. In the future, this noise can be improved by implementing a detector on the photonic chip itself, as depicted in Fig. 1a. The electronic noise floor, of ~110 pT/√Hz, was measured by turning off the laser and acquiring the output signal of the balanced photodetector. By further optimization of the geometry, mechanical stability, and overall packaging of the presented hybrid system to realize a standalone photonic–atomic chip, the sensitivity is expected to be further improved towards the photon shot-noise limit.

## Discussion

As previously stated, the magnetic sensitivity is inversely proportional to the atomic volume to be probed, especially in our 30 μm-long cell, where the dominant atomic spin destruction process is wall collisions. Following the discussion presented in ref. ^16^, for a given volume, *V*, optimizing the atomic density would result in spin-collision-limited magnetometers, in which the spin-collision rate is linearly proportional to the atomic density, *ρ*. In this case, the fundamental quantum-mechanical-limited magnetic sensitivity can be rewritten as:

2 $$\delta B_{\mathrm{ASN}} \approx \frac{1}{\gamma }\sqrt {\frac{\xi }{{V\tau }}}$$

where *V* is the volume of interacting atoms and *ξ* is the relaxation constant such that Γ = *ξ·ρ* and the number of atoms in the interaction volume is *N* = *ρ·V*. Thus, the fundamental sensitivity scales as 1/√*V*, and for a volume of 1 μm^3^, a sensitivity of ~1 nT/√Hz is expected. In our case, the interaction volume is 30 × 3.2 × 3.2 μm^3^, and the expected fundamental sensitivity (without coatings) is limited to ~60 pT/√Hz. These numbers can be further improved by adding buffer gas, thus reducing the atomic spin-relaxation rate due to wall collisions^18^ (Supplementary Information 3).

The main advantage of our demonstrated platform is the flexibility offered by integrating a microfabricated alkali vapor cell with a highly configurable photonic chip. Using this platform, a large range of spatial resolutions and magnetic sensitivities can be designed and implemented. For example, for applications requiring higher sensitivity, a millimeter-scale Rb cell can be easily implemented. Such cells have been shown to produce sensitivities as low as 5 fT/√Hz^25^, and they can be integrated on top of a chip-scale device, following the concept shown in ref. ^34^. In this case, the magnetic field in the longitudinal, *z*, the direction will be averaged, and the in-plane spatial resolution, *x*–*y*, can still range within the micrometer scale.

In conclusion, we have demonstrated the design, fabrication, and experimental characterization of a chip-scale hybrid nanophotonic-atomic quantum technology based on a micrometer vapor cell implemented on a photonic chip for the purpose of high-resolution and sensitive magnetometry. The magnetometer is based on the NMOR method, in which the magnetic field is sensed by recording the change in the state of polarization of the light interacting with the atoms. The change in the ellipticity of polarization of the probe beam is analyzed and measured using an inverse designed photonic spin selector, which spatially resolves the incident photons depending on their handedness. A magnetic sensitivity of 700 pT/√Hz was measured for an interaction volume of ~300 μm^3^. The flexibility offered by the presented hybrid platform enables future development of high-resolution and sensitive magnetic imaging techniques, with the vision of constructing a magnetic image sensor array based on this technology.

Ultimately, further improvements could enable the development of integrated micrometer-scale spatial resolution magnetometers with sensitivity down to the pT/√Hz range by using approaches such as adding buffer gas and adding antirelaxation coatings, which enable non-destructive atomic spin wall collisions. To implement the latter technology in integrated microfabricated alkali vapor chips, one needs to develop coating materials that can sustain high temperatures or develop low-temperature bonding techniques. Both approach are active research areas^35–38^.

## Materials and methods

### Optimization algorithm

The direct-binary-search (DBS) algorithm was used for the optimization of the nanophotonic structure. We designed a disk with 450 pixels and a minimal spatial resolution of 120 nm × 120 nm for convenient fabrication in electron beam lithography. Each pixel was assigned a binary number, denoted by 0 or 1, indicating whether the SiN in the pixel will be etched or not. The FOM for optimization was to maximize the coupling of right circularly polarized light incident on the top of the device to the fundamental TE mode of one waveguide and left circularly polarized light to the fundamental mode of the second waveguide (Supplementary Information 1). In the first step, defined as the initialization step, many random patterns were simulated, and the one with the highest FOM was chosen as a starting point for the second step, defined as the optimization step. In this step, the binary value of each pixel state was swapped, and the FOM was calculated. If the FOM was improved, then the new pixel state was saved, and the search moved to the next parameter. However, if no improvement was achieved, then the pixel state was swapped back to its original value. After each iteration, the new FOM was compared with the previous FOM, and the algorithm was stopped when the FOM reached the desired value. The implementation of the first step is important, as it was found to reduce the probability of converging to a local minimum.

### Device fabrication

The device fabrication process of the photonic chip began with a silicon chip with a 250-nm-thick layer of stoichiometric SiN deposited by low-pressure chemical vapor deposition on top of a 2 μm-thick layer of thermal oxide. The SiN was patterned by e-beam lithography and etched by reactive ion etching (RIE) to form the inverse designed PSS and coupling waveguides. The device was then covered by a 2-μm-thick layer of silicon oxide for encapsulation. Then, a specific region of the top oxide layer on top of the PSS was removed using buffered hydrofluoric acid wet etching to expose the PSS to air since the device performs more efficiently under higher index contrast. After fabrication, the dimensions of the device were measured using a scanning electron microscope and refined in the next e-beam lithography mask to take into account and correct the proximity effect. After a few iterations, the dimensions were optimized to those used in the simulation. Further improvement of the fabrication process would be to measure the refractive index of our SiN layer and run the simulation with the newly measured parameter, as in^27^.

### Micrometer cell fabrication

Our cell consists of two glass wafers bonded together using the anodic bonding technique. First, 250-nm-thick amorphous silicon (a-Si) layer was deposited on a Borofloat 33 glass chip (16.4 mm × 16.4 mm) by plasma-enhanced chemical vapor deposition and patterned by a laser writing lithography system (UV 405 nm), to obtain the desired cell geometry (Fig. 3a). The a-Si layer was then etched by RIE, and the glass was wet-etched in an HF:H_2_O 1:10 solution to define the 30 μm Rb interaction chamber. In parallel, the Rb dispenser chamber was prepared in the second glass chip by drilling a 2 mm deep and 4 mm diameter hole by CNC. After cleaning, an Rb dispenser pill (SAES group) and the glass substrates were stacked together and inserted into a custom-made vacuum chamber for anodic bonding. The two glass substrates were placed between two graphite plates and two stainless steel electrodes. Next, the structure was heated, to 350 °C. Next, a high voltage of 700 V was applied for ~40 min. After anodic bonding was performed, Rb vapor in natural abundance was released by heating the dispenser pill using a 1.5 W diode laser at a wavelength of 830 nm for ~20 s.

### Experimental setup

An external cavity diode laser at a wavelength of 780 nm was initially prepared with linear polarization and focused from the top onto the PSS using a 5× microscope objective. The laser beam passed through our micrometer-scale Rb cell, which was heated to ~120 °C, corresponding to an Rb density of ~1.6 · 10^13^ cm^3^. Two lensed fibers were used to collect the light from the photonic chip. These fibers were connected to fiber variable optical attenuators prior to a balanced photodetector to compensate for the difference in coupling efficiency. The photonic chip with the micrometer Rb cell was placed in the center of a set of custom-made Helmholtz coils for the magnetic measurements.

## Supplementary information

Supplementary information for “Demonstration of an integrated nanophotonic chip-scale alkali vapour magnetometer using inverse design”
