# Supplementary material for: Demonstration of an integrated nanophotonic chip-scale alkali vapor magnetometer using inverse design
Source: Light Sci Appl. 2021 Mar 11;10:54. doi: 10.1038/s41377-021-00499-5 (PMC7952415; doi:10.1038/s41377-021-00499-5)
Supplement: Supplementary file 1 — Supplementary information for “Demonstration of an integrated nanophotonic chip-scale alkali vapour magnetometer using inverse design” [file 41377_2021_499_MOESM1_ESM.docx]

Supplementary information for
“Demonstration of an integrated nanophotonic chip-scale alkali vapour magnetometer using inverse design”

*Yoel Sebbag^†^, Eliran Talker^†^, Alex Naiman, Yefim Barash & Uriel Levy^*^*

*† these authors equally contributed to this work*

** corresponding author:* [*ulevy@mail.huji.ac.il*](mailto:ulevy@mail.huji.ac.il)

**Department of Applied Physics, the Center for Nanoscience and Nanotechnology, the Hebrew University of Jerusalem, Jerusalem, 91904, Israel.**

1. **Inverse design simulations**
   1. **Structure design**

The structure of the photonic spin selector (PSS) consists of a disk with radius of 1.8 μm. The disc is sub-divided in 15 rings of 120 nm width. Each ring is divided in pixels with azimuthal width ranging from 120 nm to 190 nm., as depicted in Figure S1. The final device is composed of 450 pixels. Each pixel was associated to a binary number, “0” or “1”, that indicates if the pixel is either filled with air or with material (silicon nitride).


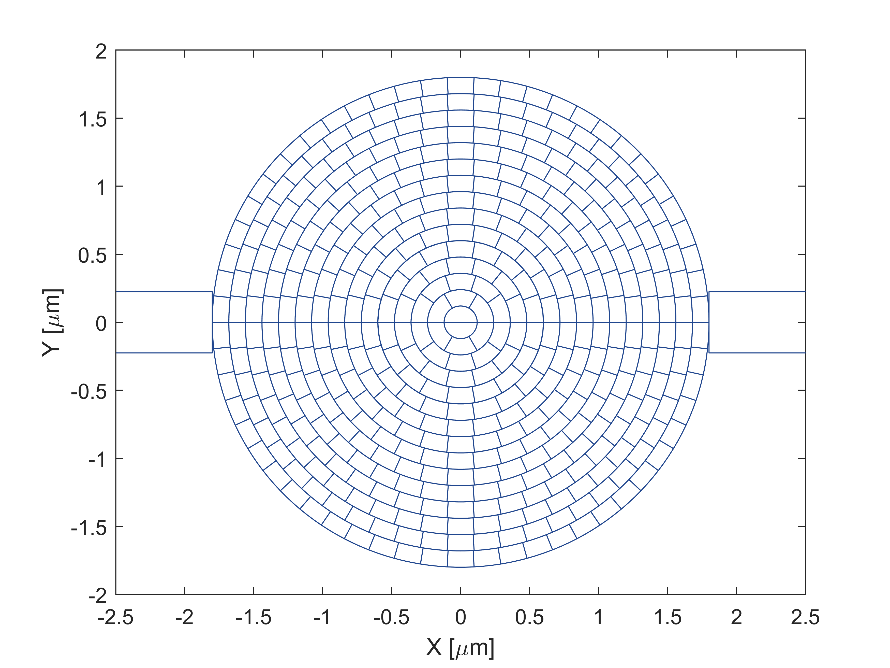


**Figure S1. Structure design.** Geometry of the PSS with radius of 1.8 μm and 450 pixels.

- 1. **Simulations**

The figure of merit (FOM) was defined as

$$FOM = 0.5*\frac{T_{L}^{RCP}-T_{R}^{RCP}}{T_{L}^{RCP}}+ 0.5*\frac{T_{R}^{LCP}-T_{L}^{LCP}}{T_{R}^{LCP}}$$

Where $T_{L}^{RCP}$ and $T_{R}^{RCP}$ are the power transmitted in the left and right waveguides respectively, in the case of right circularly polarized light incident on the device. Similarly, $T_{L}^{LCP}$ and $T_{R}^{LCP}$ are the power transmitted in the left and right waveguides respectively, for the case of incident left circularly polarized light. The optimization algorithm was meant to maximize this FOM.

The optimization procedure was composed of 3 steps.

**The initialization step:** In this first step, the device was simulated about 500 times, with random patterns. The one presenting the highest FOM was selected for the second step.

**The optimization step:** In this step, each pixel is swapped one after the other, and the FOM is calculated. If the FOM is improved, the new binary value associated with the pixel is saved, otherwise, the pixel value is swapped back to its original value. Then, the algorithm moves to the next pixel. One iteration ends when all the 450 pixels were tested. the algorithm moves to the third step, in which

**The finalization step:** In this third step, the FOM was calculated and it is compared to value of the previous iteration. The algorithm stops if one of the following conditions was fulfilled:

1. The maximum iteration number was reached
2. The FOM at the end of the current iteration is lower than the previous one.
3. If the relative change in the FOM is smaller than 2% with respect to the one from the previous iteration (which was found to be a good compromise between performance and computation time)

If none of the conditions is fulfilled, the algorithm goes back to step two. A typical FOM graph as function of iterations steps and for each iteration are presented in Figure S2.

In this work, we did not optimize the coupling to a specific waveguide mode since we are interested in the total energy transmitted to the left or right waveguide. However, more that 90% of the coupled energy was carried by the fundamental TE mode of the waveguide.

Also, we did not optimize the coupling efficiency from free space to the waveguide mode. It was about 1.5 %, which is enough for the proof of concept and magnetic field measurement, however, it can be obviously improved, for energy consumption point of view.

It should be noted that the simulations were performed using an incident plane wave. In the future, further optimization of the presented device could include a figure of merit function considering the tolerance to possible optical misalignment.


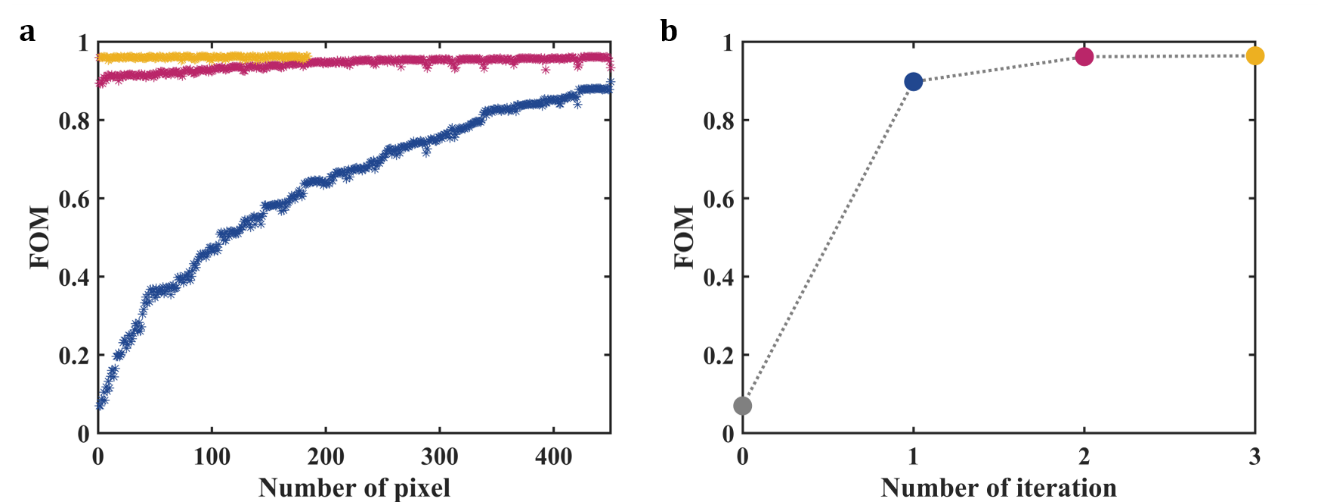


**Figure S2. Typical convergence of the algorithm.** Calculated FOM as function of the number of pixel swaps (a), and at the end of each iteration (b).

- 1. **Fabrication**

Description of the fabrication steps of the PSS is presented in Figure S3.

A small rib between 30 nm to 50 nm was used as a stop layer for the last step of etching the upper PECVD oxide. Simulations were run for optimizing the performances of the PSS, depending on the rib height. As an example, the obtained geometries for the case of 30 nm and 50 nm rib are presented in Figure S4.


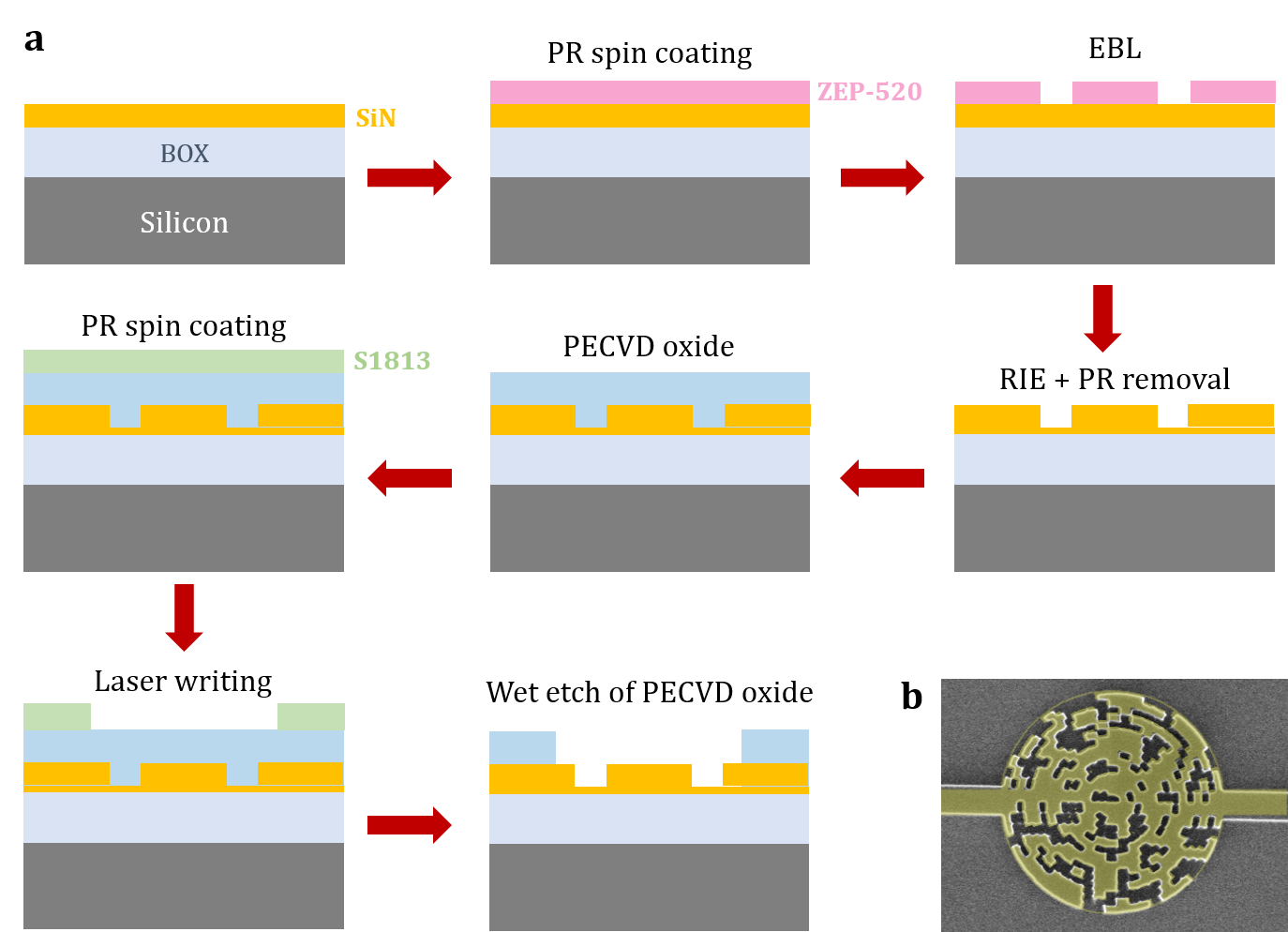


**Figure S3. Fabrication process.** (a) The device was fabricated on a silicon wafer with 250 nm of low-pressure chemical vapor deposition (LPCVD) silicon nitride (SiN), on top of 2 μm of buried thermal oxide (BOX). An electron beam lithography photoresist (PR), ZEP-520 is spin coated on top of the chip. Then, the PR is patterned by electron-beam lithography (EBL). The PR is developed, and the pattern is transferred to the SiN by reactive ion etching (RIE). After removing the PR residue, the device is covered by 2 μm of silicon oxide for encapsulation, by pressure enhanced chemical vapor deposition (PECVD). In order to expose the PSS to air, the oxide laser is etched. This is done by spin coating the device with PR (S-1813) and exposition to laser writer. The pattern in the PR is then transferred to the oxide layer by wet etch. (b) Scanning electron microscope (SEM) image of the fabricated device, with the superimposed geometry used in the simulation.


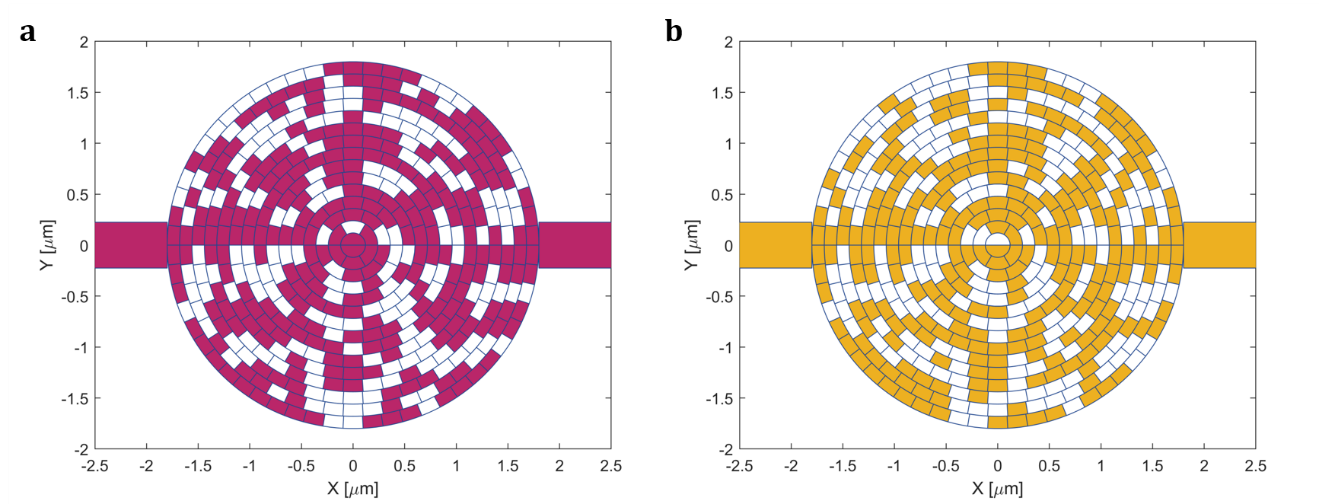


**Figure S4. Optimized geometry.** Optimized structure for the case of 30 nm rib (a), and 50 nm rib (b).

1. **Nonlinear magneto-optic rotation in micrometer Rb cell**

In linear magneto-optic rotation, also known as Faraday effect, the polarization rotation angle, $\alpha$, can be calculated using circular refractive indices $n_{+}$ and $n_{-}$, associated to the right ($\sigma_{+}$) and left ($\sigma_{\_}$) circular polarization, respectively.

$$\alpha=\frac{\omega L}{2c}\left( n_{+}-n_{-} \right)$$

where $\omega$ is the light frequency, L is the length of the medium, and c the speed of light.

In alkali vapors, the nonlinear magneto optic rotation (NMOR) occurs near resonance and, according to Kramers–Kronig relations, this magnetically induced circular birefringence is also associated to a circular dichroism. The latter effect can be measured as a change in ellipticity of the interacting light.

In order to calculate the susceptibility of rubidium (Rb) vapors, and hence the dependence of the complex refractive indices to the ambient magnetic field, we use the density matrix formalism, under the rotating-wave approximation (RWA).

Here we describes the general formalism used to calculate the NMOR in a micrometer cell, near the $5^{2}S_{1/2}\left( F=3 \right)\to5^{2}P_{3/2}(F^{'}=4)$ transition of ^85^Rb. The relevant Zeeman sub-levels are presented in Figure S5.


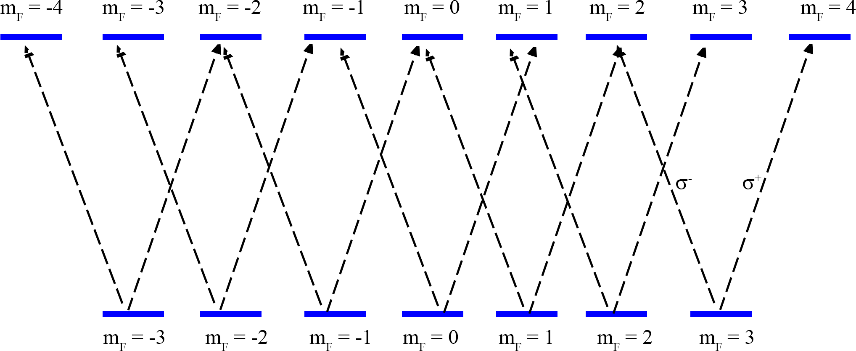


**Figure S5.** Diagram level of ^85^Rb, presenting the relevant Zeeman sub-levels of the $5^{2}S_{1/2}\left( F=3 \right)\to5^{2}P_{3/2}(F^{'}=4)$ transition.

The time evolution of the density matrix, $\rho$, is governed by the Liouville equation:

$$\dot{\rho}=-\frac{i}{\hbar}\left[ \mathcal{H,}\rho\right]-\frac{1}{2}\left\{ \Gamma,\rho\right\}$$

where $\mathcal{H}$ is the total Hamiltonian of the system and $\Gamma$ the relaxation operator.

The total Hamiltonian is the sum of the unperturbed Hamiltonian, the magnetic field interaction Hamiltonian and the light-matter interaction Hamiltonian:

$$\mathcal{H=}\mathcal{H}_{0}+\mathcal{H}_{B}+\mathcal{H}_{L}$$

- The unperturbed Hamiltonian, $\mathcal{H}_{0}$, under RWA, is defined as:

$$\mathcal{H}_{0}=\hbar\sum_{m_{F}=-4}^{m_{F}=4} \Delta_{pr}\cdot\left| F_{e}=4,m_{F} \right\rangle\left\langle F_{e}=4,m_{F} \right|$$

were $\Delta_{pr}$ is the frequency detuning from the resonance of the probe beam.

- We describe the interaction with the magnetic field, with the Hamiltonian, $\mathcal{H}_{B}$, in which we consider a magnetic field, $B_{z}$, aligned to the quantization axis in the $\hat{z}$-directed. Under the RWA, this Hamiltonian is given by:

$$\mathcal{H}_{B}=g_{F}\mu_{B}B_{z}F_{z}$$

$$\mathcal{H}_{B}=g_{F}\mu_{B}B_{z}\left( \sum_{m_{F}=-3}^{m_{F}=3} m_{F}\left| F_{g}=3,m_{F} \right\rangle\left\langle F_{g}=3,m_{F} \right|+\sum_{m_{F}=-4}^{m_{F}=4} m_{F}\left| F_{g}=4,m_{F} \right\rangle\left\langle F_{g}=4,m_{F} \right| \right)$$

where $F_{z}$ is the macroscopic angular momentum of the atoms in the hyperfine states, and it is equal to $F_{z}\left| m_{F} \right\rangle=m_{F}\left| m_{F} \right\rangle$.

- The light-atom interaction action Hamiltonian, also the under the RWA, is equal to:

$$\mathcal{H}_{L}=\frac{1}{2}\Omega_{pr}\sum_{F=2}^{3} \sum_{F^{'}=F-1}^{F+1} \sum_{m=-F}^{F} C_{F,m}^{F^{'},m+1}\left| F,m \right\rangle\left\langle F^{'},m+1 \right|+\frac{1}{2}\Omega_{pr}\sum_{F=2}^{3} \sum_{F^{'}=F-1}^{F+1} \sum_{m=-F}^{F} C_{F,m}^{F^{'},m-1}\left| F,m \right\rangle\left\langle F^{'},m-1 \right|+H.C.$$

where $\Omega_{pr}$ is the Rabi frequency of the probe beam and $C_{F,m}^{F^{'},m+1}$ are the normalized transition coefficients  [1]. The first term in the right-hand side of the equation describes the interaction with right circularly polarized light ($\sigma^{+}$) while the second term is related to the interaction with left circular polarization ($\sigma^{-}$). H.C. stands for Hermitian conjugate.

The relaxation matrix, $\Gamma$, in the Liouville equation given by:

$$\Gamma=(\gamma_{0}+\gamma)\sum_{m_{F}=-4}^{4} \left| F=4,m_{F} \right\rangle\left\langle F=4, m_{F} \right|$$

Here, we assume that the excited state spontaneously decays to the ground state with rate $\gamma_{0}$, and the ground state relaxes with rate $\gamma$. This later relaxation is partially due to the “transit” effect, in which the atoms exit area of interaction defined by the light beam. The rate $\gamma$ is also related to the collisions rate, atom-atom collisions as well as collisions with the walls of the cell. This rate is defined as:

$$\gamma=\frac{\bar{v}}{\bar{l}}+\frac{v_{z}}{L/2}$$

where $\bar{v}$ is the mean atomic velocity, $\bar{l}$ is the mean free path, $v_{z}$ is the atomic velocity in the direction perpendicular to the cell walls, and L is the cell length. The mean free path is defined as $\bar{l}=\bar{v}/\Gamma_{SE}$, where $\Gamma_{SE}$ is the spin exchange rate.

The susceptibility of the atomic medium is then liked to the density matrix elements according to  [2]

$$\chi_{\pm}=\frac{N}{E_{0}}\sum_{m=-3}^{3} d_{m,m\pm1}\rho_{m\pm1,m}$$

where $\rho_{m\pm1,m}$ is the optical coherence between the ground state sublevel m and excited state sublevel $m\pm1$ and $d_{m,m\pm1}$ is the corresponding dipole matrix elements, N is the density of the atoms, and E_0_ is the amplitude of the electric field of the light.

$$\rho_{m,m^{'}}=\left| m \right\rangle\left\langle m^{'} \right|$$

$$d_{m,m^{'}}=C_{m}^{m'}\cdot d$$

where $C_{m}^{m'}$ is a coefficient that determines the transition strength of a particular transition, and is dependent on the initial and final states of the transition, and$d = 5.177\cdot e\cdot a_{0}$ (with e – electron charge, a_0_ – the Bohr radius). The nonlinear magneto-optic rotation angle and its associated circular dichroism can be computed using the density matrix elements. They are given by:

$$\alpha=\frac{\omega LN}{2cE_{0}}Re\left\{ \sum_{m=-3}^{3} d_{m,m+1}\rho_{m+1,m}+\sum_{m=-2}^{3} d_{m,m-1}\rho_{m-1,m} \right\}$$

$$\varepsilon=\frac{\omega LN}{2cE_{0}}Im\left\{ \sum_{m=-3}^{3} d_{m,m+1}\rho_{m+1,m}-\sum_{m=-2}^{3} d_{m,m-1}\rho_{m-1,m} \right\}$$

The calculated NMOR in a $30 \mu m$ long Rb cell, interacting with a light beam with intensity of about $4.8 mW/cm^{2}$ is presented in Figure S6. If there is no additional buffer gas in the cell (Figure S6.a), the NMOR width is limited by wall collisions. This graph is in good agreement with the measured NMOR in Figure 5.b of the main article. By adding buffer gas, the ground state relaxation rate decreases. In Figure S6.b, the NMOR is calculated in the case of addition of about 300 Torr of N_2_. In that case, the relaxation rate is about $2\pi\cdot1 GHz$  [3]. It can be seen from this graph that the width of the NMOR is narrower and the slope of the linear part is higher, leading to increased magnetic sensitivity.


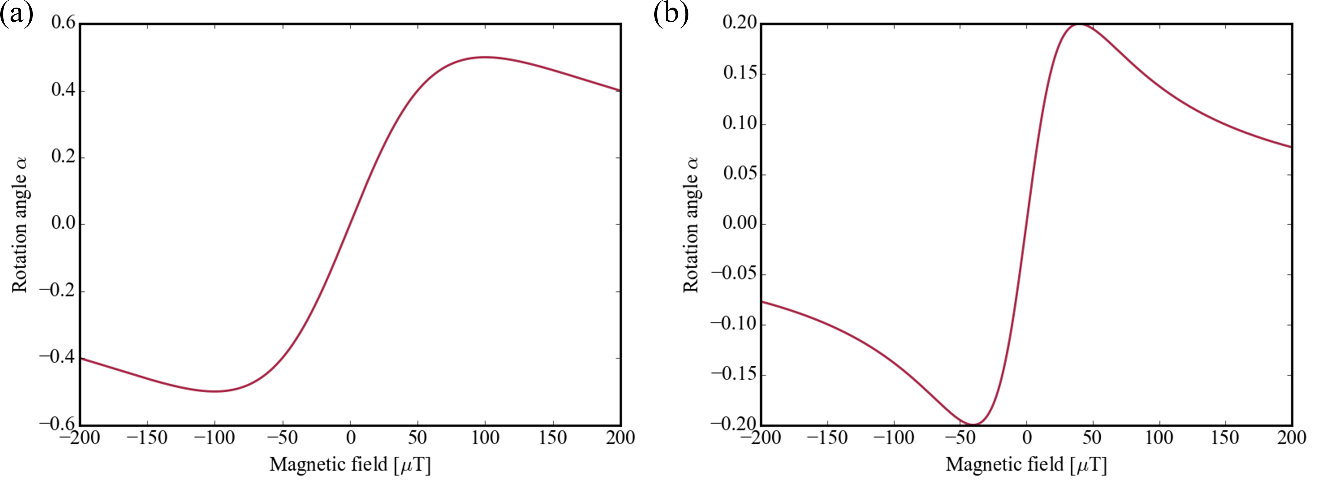


**Figure S6. NMOR in micrometer Rb cell.** Calculated NMOR in a 30 μm long Rb cell, at light intensity of $4.8 mW/cm^{2}$, without any additional buffer gas (a), and with the addition of about 300 Torr of N_2_ (b).

1. **Magnetic sensitivity**

In our custom 30 µm Rb cell, without any additional buffer gas, the spin relaxation rate, $\Gamma$, is mainly influenced by spin destruction collision – with rate $\Gamma_{SD}$ – and the wall collision, described by the rate $\Gamma_{wall}$:

$$\Gamma=\Gamma_{SD}+\Gamma_{wall}$$

The spin destruction rate is linearly proportional to the mean velocity of the atoms $\bar{v}$, and the density of Rb atoms, $\rho$  [4]:

$$\Gamma_{SD}=\sigma_{Rb}^{SD}\cdot\bar{v}\cdot\rho$$

where $\sigma_{Rb}^{SD}$ is the spin-destruction cross section. For the case of Rb atoms $\sigma_{Rb}^{SD}=9\times{10}^{-18} cm^{2}$.

Moreover, the wall collision rate can be described as [5]:

$$\Gamma_{wall}=\frac{\bar{v}\cdot S}{4\cdot V}$$

where V and S are the volume of the cell and surface area, respectively.

In both cases, the most probable velocity of the Rb atoms is:

$$\bar{v}=\sqrt{\frac{8\cdot k_{B}\cdot T}{\pi\cdot m}}$$

where $k_{B}$ is the Boltzmann constant, T is the temperature of the cell and $m$ is the mass of the Rb atoms.

Since the atomic density also depends on temperature [6], the predicted magnetic sensitivity presented in equation (1) can be plotted as function of the Rb cell’s temperature. This sensitivity is presented in Figure S7. It can be seen that in the first part of the graph, i.e. the regime dominated by atom-wall collision, the ratio $\sqrt{\Gamma/N}$ is within one order of magnitude higher than the minimum, reached at about 300°C.

Although the optimal sensitivity is reached at a temperature of about 300°C, in this work, we have chosen to operate our magnetometer at a relatively low temperature of 120°C to avoid Rb reaction to the Borosilicate cell’s walls, typically operated up to 150°C [7]. In the future, a cell fabricated with different glass, such as sapphire, may enable operation up to 200°C [8].


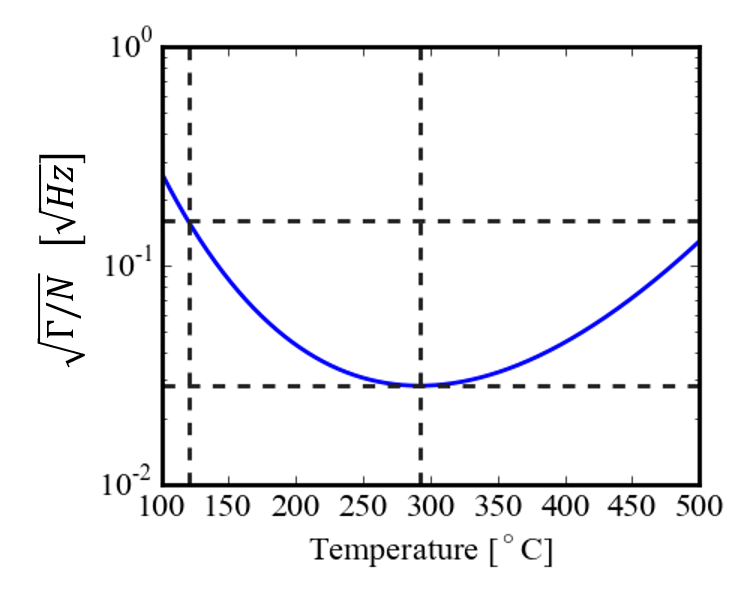


**Figure S7 Magnetic sensitivity.** The graph shows the ratio of $\sqrt{\Gamma/N}$ as function of the Rb cell’s temperature. This corresponds to the fundamental magnetic sensitivity limit presented in equation (1).

**References:**

[1] P. Siddons, C. S. Adams, C. Ge, and I. G. Hughes, J. Phys. B At. Mol. Opt. Phys. **41**, (2008).

[2] Y. P. Malakyan, S. M. Rochester, D. Budker, D. F. Kimball, and V. V. Yashchuk, Phys. Rev. A **69**, 013817 (2004).

[3] P. J. Oreto, Y.-Y. Jau, A. B. Post, N. N. Kuzma, and W. Happer, Phys. Rev. A **69**, 042716 (2004).

[4] J. C. Allred, R. N. Lyman, T. W. Kornack, and M. V. Romalis, Phys. Rev. Lett. **89**, 1308011 (2002).

[5] A. Aleksanyan, S. Shmavonyan, E. Gazazyan, A. Khanbekyan, H. Azizbekyan, M. Movsisyan, and A. Papoyan, J. Opt. Soc. Am. B **37**, 203 (2020).

[6] C. B. Alcock, V. P. Itkin, and M. K. Horrigan, Can. Metall. Q. **23**, 309 (1984).

[7] A. WHITTAKER, KATE, Construction and Characterisation of Ultra-Thin Alkali-Metal Vapour Cells, 2017.

[8] N. Sekiguchi, T. Sato, K. Ishikawa, and A. Hatakeyama, Appl. Opt. **57**, 52 (2018).
